# Supplementary material for: CAF-Associated Genes in Breast Cancer for Novel Therapeutic Strategies
Source: Biomedicines. 2024 Aug 29;12(9):1964. doi: 10.3390/biomedicines12091964 (PMC11428270; doi:10.3390/biomedicines12091964)
Supplement: Supplementary file 1 [file biomedicines-12-01964-s001.zip › biomedicines-3098792-supplementary.pdf]

Figure S1

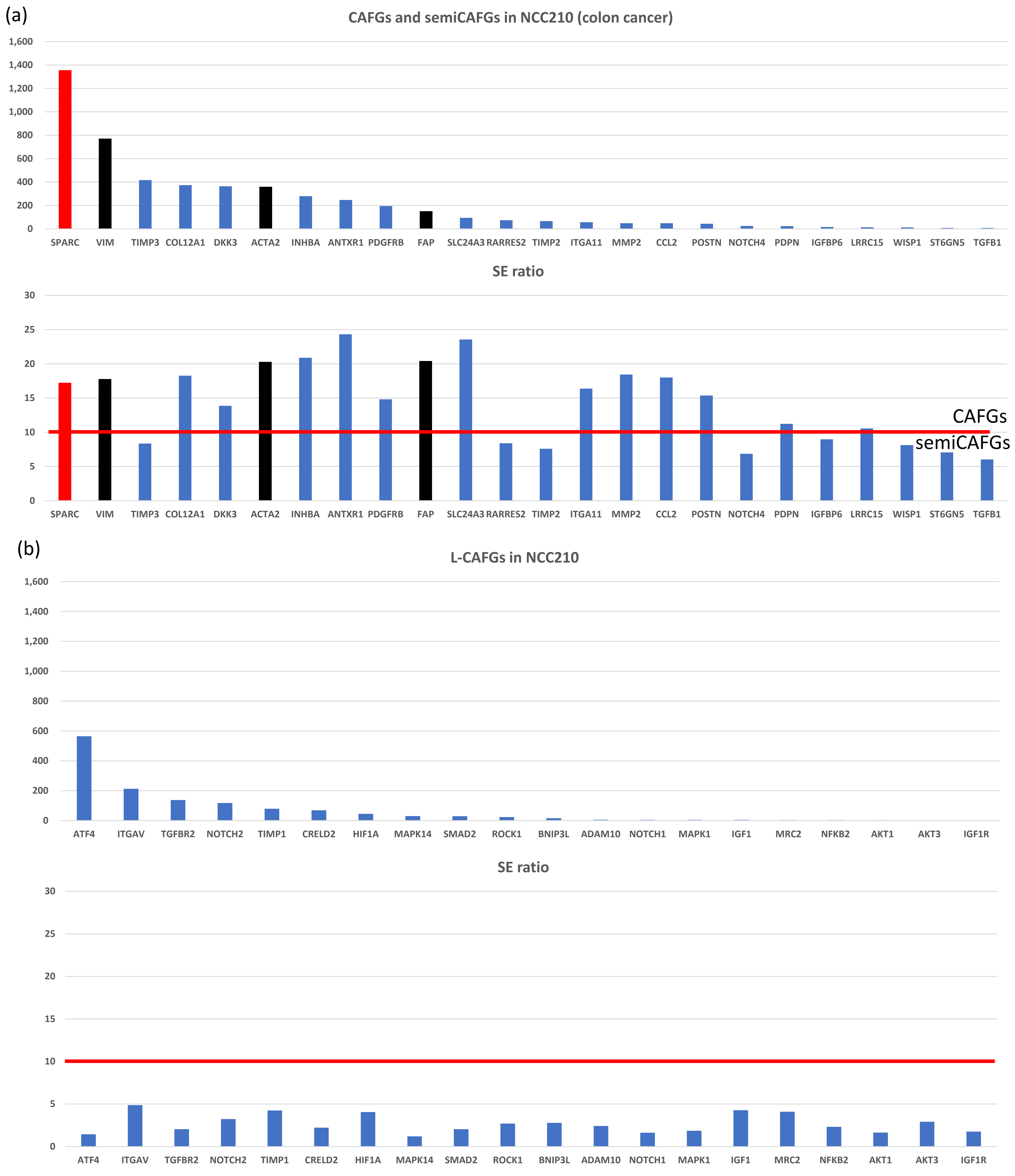

(a)
CAFGs and semiCAFGs in NCC210 (colon cancer)
1,600
1,400
1,200
1,000
800
600
400
200
0
SPARC VIM TIMP3 COL12A1 DKK3 ACTA2 INHBA ANTXR1 PDGFRB FAP SLC24A3 RARRES2 TIMP2 ITGA11 MMP2 CCL2 POSTN NOTCH4 PDPN IGFBP6 LRRC15 WISP1 ST6GN5 TGFB1
SE ratio
30
25
20
15
10
5
0
CAFGs
semiCAFGs
SPARC VIM TIMP3 COL12A1 DKK3 ACTA2 INHBA ANTXR1 PDGFRB FAP SLC24A3 RARRES2 TIMP2 ITGA11 MMP2 CCL2 POSTN NOTCH4 PDPN IGFBP6 LRRC15 WISP1 ST6GN5 TGFB1
(b)
L-CAFGs in NCC210
1,600
1,400
1,200
1,000
800
600
400
200
0
ATF4 ITGAV TGFBR2 NOTCH2 TIMP1 CRELD2 HIF1A MAPK14 SMAD2 ROCK1 BNIP3L ADAM10 NOTCH1 MAPK1 IGF1 MRC2 NFKB2 AKT1 AKT3 IGF1R
SE ratio
30
25
20
15
10
5
0
ATF4 ITGAV TGFBR2 NOTCH2 TIMP1 CRELD2 HIF1A MAPK14 SMAD2 ROCK1 BNIP3L ADAM10 NOTCH1 MAPK1 IGF1 MRC2 NFKB2 AKT1 AKT3 IGF1R

Figure S1. Semi-CAFGs and L-CAFGs in CRC

(a) Upper panel, expression amounts of CAFGs and Semi-CAFGs in NCC210 (colon cancer). Lower panel, SE ratio of the corresponding upper panel. Black bars are CAFs markers.

(b) Upper panel, expression amounts of L-CAFGs in NCC210 (colon cancer). Lower panel, SE ratio of the corresponding upper panel.

SYMBOL	CAFG	GSM2726	GSM2726	GSM2726	GSM2726	GSM2726	GSM2726	GSM2726	GSM2726	GSM2726	GSM2726	GSM2726	GSM2726	GSM2726	GSM2726
	SPARC	138.792	830.566	1459.65	808.765	2049.94	1852.97	53.2985	534.384	808.411	1946.27	115.687	249.308	3611.75	3951.23
1	POSTN	706.792	4978.72	1921.31	1185.96	5111.4	2453.41	60.8971	2069.42	1848.05	3557.9	1134.47	1193.97	6763.15	6441.28
2	ASPN	2.14894	1152.8	1234.22	552.756	1723.22	2194.26	77.4649	43.5265	532.739	1714.93	138.565	29.6848	1120.54	3615.91
3	COL1A2	395.733	2692.23	2337.74	2785.32	5050.7	6533.38	76.424	1259.2	3515.96	5356.04	195.437	1001.77	4797.03	10223.5
4	COL5A2	7.84293	143.883	237.886	151.38	291.136	471.91	11.7741	73.7938	118.253	478.051	24.0613	83.2237	521.914	850.143
5	AEBP1	72.6511	181.139	240.322	238.344	207.814	365.632	8.14495	91.1598	46.9911	196.291	35.4562	111.813	499.816	318.197
6	COL5A1	18.9187	367.828	540.005	347.969	718.195	654.362	5.11452	133.192	12.9934	59.3401	10.4043	172.412	692.687	1735.3
7	COL3A1	173.668	62.5105	751.178	639.727	1412.38	739.947	45.1447	515.311	94.2451	583.804	5.8434	130.225	2964.73	1022.88
8	THBS2	448.633	4463.65	2958.49	2479.69	6088.38	5368.68	31.2876	1272.17	1894.36	12739.9	1596.79	3628.68	4267.19	9343.29
9	VCAN	12.9503	64.2514	69.2483	103.494	142.756	302.446	8.00834	59.6191	54.3762	271.125	22.638	94.3993	113.31	470.981
10	LUM	447.595	5030.47	1256.43	1534.46	3235.39	6090.52	110.268	1467.89	548.956	8254.13	1014.29	3660.96	3468.2	7086.48
11	FSTL1	18.8614	284.943	110.8	114.208	268.522	171.531	5.34624	33.5778	44.7613	194.836	8.43912	111.498	147.017	435.265
12	DKK3	67.1838	99.7858	575.064	177.646	138.316	198.473	3.95461	13.1252	22.1014	943.054	7.7985	27.0135	204.3	1353.45
13	DCN	97.2932	1252.36	1014.32	602.708	1864.04	923.284	205.835	255.66	685.936	3414.13	226.21	1278.44	655.862	1463.81
14	SPON1	23.2273	29.8144	219.47	240.406	190.18	816.873	11.8403	33.4938	72.1309	723.289	27.0218	56.58	161.208	412.075
15	FBLN1	67.6586	1201.3	896.678	795.756	1215.28	1531.3	211.334	327.782	722.311	3328.86	430.076	328.991	659.18	1550.1
16	MMP2	26.5499	435.069	279.55	298.801	419.64	655.443	21.5042	137.552	217.473	1099.84	96.9841	244.488	196.921	691.5
17	COL6A3	139.4	1901.83	912.905	982.063	2163.21	2904.51	240.213	674.782	562.958	4790.18	491.98	1421.59	2062.66	2405.08
18	MXRA5	77.068	179.806	532.437	441.527	783.566	484.212	44.0686	81.9437	165.771	1201.32	11.6329	29.862	1172.44	1794.82
19	PALLD	160.992	703.173	824.852	487.661	967.576	701.501	5.66615	192.53	127.183	4079.36	34.611	276.285	1452.42	2242.25
20	CALD1	65.4399	300.025	233.205	89.0232	326.993	227.285	8.76498	48.8847	40.8625	362.291	16.3526	64.1877	373.917	1141.51
21	NR3C1	4.07332	42.835	70.401	35.2797	26.0836	145.329	2.42097	3.7501	38.9425	45.2122	6.09453	44.3506	94.9154	80.4044
22	ISLR	6.55384	29.5203	17.9429	11.6505	19.6732	19.9031	3.22349	7.92402	18.7855	27.2456	10.4572	25.845	20.7721	53.2646
23	HOPX	2.03886	14.9535	20.757	8.06013	14.4977	12.2695	2.77391	3.26082	17.2132	21.8309	7.91034	34.5613	21.0563	16.1279
24	LOC1019	294.915	1848.29	1126.33	1151.04	3898.45	1516.07	8.91395	254.428	52.5455	2752.72	21.7682	1183.3	122.3	3673.54
25	SERPINF1	2.77483	21.0937	28.4076	16.0912	12.5143	23.8487	2.57291	11.5815	21.8995	43.531	8.96869	29.8852	13.5085	28.455
26	ITIH5	2.88769	21.7447	5.34109	26.0419	16.4925	16.7922	2.11548	5.48364	25.9329	46.6035	17.8742	30.9028	14.7742	48.7339
27	COL1A1	174.362	1289.48	1569.74	1164.07	2338.71	2255.15	62.3553	612.575	965.937	1312.42	27.3279	258.26	2561.32	2376.86
28	CDH11	8.61793	347.116	66.2217	44.2582	102.09	67.5068	3.23226	89.044	13.664	52.4394	16.3552	76.9157	363.486	185.983
29	CTSK	3.52121	287.67	184.368	75.1925	198.922	135.017	4.21273	24.2891	25.6437	632.472	23.5512	28.3682	103.029	343.952
30	LAMA4	51.7098	80.2129	108.468	83.3497	279.446	23.4004	5.14959	13.3972	27.7861	159.804	7.5495	50.0628	95.2543	205.302
31	PDLIM3	2.41998	17.3548	3.95787	3.86319	10.5641	11.8174	13.6291	3.65609	10.6588	29.454	7.84026	36.5261	6.09318	15.2414
32	ACTA2	30.1184	383.991	404.529	254.616	855.573	987.273	3.51298	30.7861	135.083	330.283	18.9148	95.9576	192.522	815.171
33	MXRA8	8.56236	33.7252	53.2226	56.3354	43.7463	33.3805	5.48809	7.2006	25.7047	32.7528	9.98405	61.655	27.4269	62.0002
34	NID2	16.2193	141.191	79.8176	14.3832	20.6282	97.5667	2.66075	5.1145	21.6713	28.3051	45.9029	28.2864	41.0588	23.9659
35	BASP1	160.207	317.835	200.361	246.775	655.733	1237.05	35.8033	256.887	528.169	702.676	214.657	361.122	322.42	473.695
36	IGFBP7	1.98032	13.2357	5.41969	5.43339	9.31215	11.4295	1.52542	2.73045	11.9889	19.2129	5.85572	20.0953	22.6035	13.2364
37	LHFP	6.93449	91.517	68.6689	78.5932	212.982	337.29	3.66495	41.4502	27.6244	838.999	14.7266	60.2978	186.975	166.222
38	COL8A1	2.10308	19.2054	3.72812	4.46686	12.3409	11.8027	2.46698	3.32787	10.6942	28.1169	11.2499	19.1989	6.69219	16.2314
39	FZD1	2.19522	18.9179	3.2606	5.01884	11.7316	14.0741	1.8127	3.84922	14.0194	19.8632	6.11909	22.5151	5.67371	15.3604
40	PTRF	2.22572	18.9448	3.42584	4.13204	10.3332	11.8361	1.91172	4.30974	13.0887	16.8511	8.08864	24.3681	5.59316	19.3175
41	DPYSL3	3.66421	38.9232	34.1195	11.4814	20.6356	28.7306	3.10644	7.32059	19.0604	31.7044	15.911	36.6871	16.0869	30.4052
42	LOXL1	2.71389	18.5752	3.71498	4.28328	11.4326	14.167	2.0511	4.81705	12.3532	21.1119	6.53427	21.2763	72.2967	38.9369
43	PLEKHO1	4.06097	16.9225	3.97141	8.15146	9.76133	13.7055	1.68927	7.29678	13.4451	24.3371	6.57235	24.9449	7.2558	15.7956
44	CNN1	2.09591	18.0913	3.36867	4.6671	18.9308	11.5904	1.61417	3.58353	12.758	15.9076	5.00553	22.028	6.16138	17.4026
45	EMILIN1	2.17189	14.5463	3.22227	4.22108	9.04713	9.52863	1.80884	3.53195	10.7561	16.4197	5.73107	19.1522	5.88819	13.623
46	GPR124	2.17519	14.4463	3.98855	4.11229	11.0971	12.185	1.77293	3.94635	12.9859	18.127	6.21016	23.8656	7.8169	13.7378
47	SURF1	11.1668	26.4123	4.07276	5.40779	10.4669	16.8883	1.91605	5.87322	12.7762	22.7692	20.303	22.8471	7.19844	17.2299
48	AIF1	1.98642	16.0792	3.2669	4.58807	9.03277	12.9793	1.50555	3.33081	12.2027	19.1698	5.9141	16.3848	5.78935	14.5715
49	BCAT1	2.11354	17.3548	3.20596	4.42945	8.35627	14.9146	1.84184	10.4031	11.4301	23.3099	6.11564	26.8362	6.61351	20.0505
50	COL8A2	4.13716	21.1407	33.1335	10.8666	10.3183	17.1148	2.23172	4.61162	18.443	27.6591	9.62649	24.2473	12.1451	34.2756
51	ANTXR1	9.27144	21.3815	3.83778	5.26337	10.5175	28.4435	2.16607	4.14413	14.8881	37.7581	13.5868	32.7668	9.36437	23.3648
52	C10orf10	3.00695	22.6863	4.66245	7.70931	12.4884	13.0597	2.14389	21.2752	19.0253	33.5748	10.3089	35.0595	9.47047	21.2485
53	RECK	489.508	5669.53	502.103	1003.38	2325.47	2599.51	589.173	902.854	3408.34	6131.49	2154.41	10779.3	1552.41	4885.77
54	TSPAN4	5.52122	34.9407	7.34794	10.0162	11.9121	13.4659	2.55633	5.71614	29.8254	35.9691	7.3254	30.6468	10.5799	24.6936
55	SDC2	4.4634	19.8592	3.3056	4.29594	10.3385	15.8056	1.50141	3.8749	13.3922	20.9327	7.7421	26.8153	8.86011	14.3838
56	EFEMP1	2.3392	61.9709	6.6815	6.84037	80.8823	13.0597	2.56178	3.9125	20.794	148.075	9.80135	40.4112	8.47824	34.9988
57	SLC24A3	2.93574	17.23	3.31584	7.28401	8.53037	15.9472	2.36172	3.76583	13.5187	23.8536	12.8555	18.9242	14.0953	86.1141
58	FRZB	1.91768	17.8794	3.37763	4.31811	12.5143	12.6328	3.21668	10.8723	14.176	19.8555	7.48946	28.0376	7.47514	13.875

SYMBOL	CAFG	GSM2727	GSM2727	GSM2727	GSM2727	GSM2727	GSM2727	GSM2727	GSM2727	GSM2727	GSM2727	GSM2727	GSM2727	GSM2727	GSM2727	epi.averag	str.averag	S/E	R	
	SPARC	1252.5	2461.16	1479.4	1488.71	1941.75	2306.93	2566.99	1270.81	174.585	115.368	7805.87	651.703	1199.49	2873.61	474.245	1642.85	3.46415	1	SPARC
1	POSTN	1238.05	5030.59	1761.7	3813.04	1980.02	5438.11	2454.22	2057.94	343.695	159.126	16505	1674.25	2338.15	5732.87	902.657	3212.63	3.55908	0.93614	POSTN
2	ASPN	5.44046	1391.05	305.576	223.654	762.456	1165.99	1828.38	55.5332	7.0581	21.2118	6707.01	169.68	292.242	1747.52	233.809	1029.13	4.40158	0.92111	ASPN
3	COL1A2	2211.98	3653.18	3170.11	2135.48	2619.77	7872.89	3957.36	1862.18	451.645	245.444	28732.2	459.268	2828.94	4087.25	826.751	3946.72	4.77377	0.91917	COL1A2
4	COL5A2	175.867	247.814	202.309	209.605	131.755	223.896	216.495	70.9429	31.3435	22.1068	1360.29	17.8091	256.082	42.0605	56.9283	238.344	4.18674	0.90178	COL5A2
5	AEBP1	20.7476	395.851	255.974	592.646	430.093	437.654	491.931	178.343	78.13	43.6959	1916.19	158.994	348.993	651.411	65.3205	307.658	4.70997	0.90106	AEBP1
6	COL5A1	26.8476	282.381	715.32	449.318	584.105	198.589	1112.4	64.9131	53.4244	23.8865	3269.2	39.4262	180.855	2039.85	87.4874	518.187	5.92299	0.88678	COL5A1
7	COL3A1	240.066	2018.25	1976.1	1072.55	838.405	1161.29	1640.5	383.924	116.916	88.75	4572.05	249.462	784.323	628.06	264.332	889.723	3.36592	0.87929	COL3A1
8	THBS2	3224.08	4536.98	3320.37	3351.23	3718.72	4418.47	4313.85	6567.38	717.221	279.25	28163.1	378.944	3216.84	4990.09	851.891	4563.49	5.35689	0.87174	THBS2
9	VCAN	58.9287	141.316	138.224	77.2708	103.26	122.247	149.75	317.644	15.3538	10.5497	1026.4	34.2988	52.5601	82.7791	33.2264	147.149	4.42868	0.86218	VCAN
10	LUM	2151.49	3028.47	5362.03	2461.8	2200.36	2465.98	4046.83	2618.53	486.074	147.398	21599.3	726.952	3360.67	4435.76	761.024	3510.63	4.61304	0.86128	LUM
11	FSTL1	378.687	205.095	151.18	118.678	122.499	82.8905	432.642	172.977	16.0936	17.1606	2075.65	45.2825	52.1163	33.1976	39.0227	209.063	5.35746	0.83683	FSTL1
12	DKK3	8.47724	31.3126	33.2667	182.689	88.1379	353.335	280.468	199.12	73.7565	52.5989	3187.05	32.5855	76.3938	17.8964	73.1265	301.727	4.12609	0.83633	DKK3
13	DCN	1262.89	910.39	1144.03	689.4	755.087	1644.05	2795.68	881.461	138.165	72.9428	12973	442.364	318.014	1177.26	300.499	1398.02	4.65233	0.82407	DCN
14	SPON1	27.0988	301.485	396.174	128.451	389.983	92.3891	305.041	114.341	47.0572	26.4857	2274.18	59.5757	86.5073	185.88	40.9128	266.152	6.50536	0.82326	SPON1
15	FBLN1	1251.51	808.131	1371.22	156.356	602.142	91.2158	1857.67	36.5023	39.7984	33.3835	11872.7	180.562	179.255	1523.98	204.639	1188.25	5.80658	0.82016	FBLN1
16	MMP2	334.562	204.592	302.337	110.562	139.294	685.362	265.438	67.7985	40.3745	15.9787	2646.75	46.6428	66.2467	265.265	73.4768	357.59	4.8667	0.81859	MMP2
17	COL6A3	811.553	1366.13	2265.87	1285.5	1235.9	3112.17	2251.11	989.223	202.357	64.3017	6980.02	506.045	953.798	1215.15	353.818	1603.3	4.53143	0.8177	COL6A3
18	MXRA5	349.29	782.51	1373.61	428.143	663.62	2616.58	1036.61	1253.38	112.021	77.4036	2987.24	132.536	555.998	327.672	201.934	703.467	3.48365	0.81607	MXRA5
19	PALLD	755.046	1954.06	1688.3	532.622	812.795	352.54	1128.23	1764.54	110.914	50.6634	5400.46	836.634	396.923	852.657	337.031	1031.87	3.06166	0.80355	PALLD
20	CALD1	265.005	159.858	391.951	180.903	117.629	585.362	610.173	49.4364	63.9517	43.425	940.269	35.0406	145.534	113.206	74.7194	250.017	3.34609	0.79472	CALD1
21	NR3C1	54.5268	10.3247	56.8834	28.7861	12.9972	30.4801	16.7682	27.3141	7.88949	18.305	272.506	40.5169	47.3263	15.01	36.6094	45.7045	1.24844	0.77531	NR3C1
22	ISLR	9.1307	10.8878	29.9332	11.4326	14.6038	50.7829	8.8948	29.9609	4.2093	6.93095	72.3845	9.33205	12.4806	32.8493	8.75267	20.5919	2.35265	0.77461	ISLR
23	HOPX	9.12329	7.02394	15.8095	6.16307	6.56951	40.0888	19.6349	26.3903	2.91659	1.90468	99.7076	7.15823	5.63828	13.4717	9.65967	16.3897	1.69671	0.77461	HOPX
24	LOC1019	340.671	202.816	253.648	158	226.907	52.4606	549.95	235.296	295.406	158.698	11146.8	253.469	143.874	208.817	253.733	1147.55	4.52267	0.77399	LOC10192
25	SERPINF1	13.4296	16.7308	20.0158	8.57175	10.6387	52.8385	12.8392	31.4894	4.08022	2.28426	116.653	12.1394	8.8699	18.2604	7.87709	21.2133	2.69304	0.77319	SERPINF1
26	ITIH5	20.0107	9.54908	23.1176	11.1846	20.2542	48.2337	17.2063	44.521	4.25488	1.20446	155.193	41.9907	5.30112	21.3226	8.37943	25.1809	3.00508	0.77311	ITIH5
27	COL1A1	374.988	2386.15	1504.82	1199.91	1557.32	4588.72	2616.38	649.813	169.429	55.4348	3663.26	545.997	1465.91	1668.11	421.916	1407.67	3.33638	0.77164	COL1A1
28	CDH11	31.3932	17.7673	23.8295	129.026	131.446	87.9771	82.9085	23.5259	5.49761	11.7021	653.458	11.6909	189.023	53.3736	15.6657	103.198	6.58752	0.77112	CDH11
29	CTSK	32.8056	47.9891	204.177	37.0135	59.4939	38.9059	70.0058	34.3299	14.0245	6.69468	973.001	13.0954	52.8287	519.288	20.1453	149.066	7.39955	0.76814	CTSK
30	LAMA4	305.129	31.0103	40.4918	13.9124	22.0471	108.726	53.5419	77.7681	8.49315	13.7761	659.58	10.7157	11.2676	25.2168	32.4632	91.8774	2.83021	0.76298	LAMA4
31	PDLIM3	8.12348	8.9184	21.0793	6.78106	5.52112	141.878	6.79312	26.1418	4.76825	0.78678	2163.14	10.9516	4.54003	15.1367	5.41605	92.7727	17.1292	0.75911	PDLIM3
32	ACTA2	84.5816	194.306	35.4904	208.032	147.901	172.326	167.714	46.328	16.4327	70.8811	1631.03	39.9795	82.6533	32.6075	160.162	266.736	1.66541	0.75489	ACTA2
33	MXRA8	60.9386	12.7568	30.61	10.8173	16.8339	61.04	21.1529	51.8824	4.73946	6.52129	170.936	13.6592	11.443	36.8774	14.6586	34.6926	2.3667	0.75277	MXRA8
34	NID2	10.8687	15.0194	35.3677	9.14406	19.5564	574.735	11.8894	33.0228	3.55496	10.1154	1399.57	12.7624	17.4227	21.3303	12.0689	97.8976	8.11153	0.73997	NID2
35	BASP1	266.323	445.091	742.725	250.366	260.175	1667.57	337.863	409.409	102.32	15.9822	2571.65	134.114	289.325	212.3	153.202	479.236	3.12812	0.73637	BASP1
36	IGFBP7	5.44929	7.94124	17.3765	4.71137	6.04055	27.4522	4.99606	29.093	2.73167	0.85792	50.5999	7.94008	4.1457	14.502	4.6525	11.7106	2.51706	0.73129	IGFBP7
37	LHFP	163.258	41.7171	465.033	65.5091	134.255	140.541	695.25	40.3295	11.8049	15.0349	1175.74	44.2575	71.0481	86.1808	31.396	188.782	6.01293	0.71675	LHFP
38	COL8A1	6.95096	5.8283	18.6924	6.66712	5.72738	46.0749	8.58468	32.2884	4.21841	0.68245	76.2789	8.93809	4.09036	21.3037	5.00778	14.2125	2.83809	0.7043	COL8A1
39	FZD1	8.183	7.26864	14.0123	4.73874	5.00425	36.1451	5.18514	21.7368	2.22946	0.86866	59.7953	7.68462	4.98264	32.3725	4.87223	12.6649	2.59941	0.69978	FZD1
40	PTRF	7.37846	10.1201	14.8261	4.72061	5.93605	30.1881	5.39822	21.0479	2.85368	0.82343	53.2729	10.013	4.97257	18.6411	4.61163	11.9507	2.59142	0.6967	PTRF
41	DPYSL3	29.3235	14.3842	22.4773	9.4892	16.421	58.5654	27.7887	36.2404	4.80283	3.39164	79.3623	9.77581	8.31874	28.7582	8.12187	23.1049	2.84477	0.69093	DPYSL3
42	LOXL1	5.48484	20.9287	15.0513	5.20093	5.46073	34.4926	6.59172	24.3325	3.69595	1.33225	45.2999	10.7034	7.99671	15.3897	5.84711	15.5795	2.66447	0.68774	LOXL1
43	PLEKHO1	5.01335	11.6324	11.4103	4.88817	7.81541	39.4558	23.4049	25.3187	2.60353	3.76175	52.7828	9.88567	6.0782	35.8604	5.26205	14.2079	2.70007	0.67688	PLEKHO1
44	CNN1	5.27396	8.0178	19.1843	5.23629	5.352	31.0207	5.74573	19.9873	3.09509	0.93353	47.1339	7.20395	5.36022	13.7521	4.52812	11.4465	2.52787	0.67469	CNN1
45	EMILIN1	5.42533	10.2563	13.4431	4.86294	5.10418	29.9139	5.63575	21.8833	2.67768	0.86693	41.9223	8.69469	4.24135	16.6057	4.55709	10.3993	2.28201	0.66872	EMILIN1
46	GPR124	4.55988	7.3002	18.6018	7.31117	5.18288	29.2889	5.6547	20.6678	2.45395	0.90014	47.6416	9.74127	4.91711	13.732	4.95897	11.2293	2.26444	0.66667	GPR124
47	SURF1	12.4352	8.50625	23.5177	6.61906	5.81098	49.3261	7.79152	38.3682	3.46943	0.9471	82.4703	12.1304	5.18122	33.3657	6.10443	16.9738	2.78057	0.65934	SURF1
48	AIF1	7.79324	6.9423	15.8095	4.8862	5.7333	35.4978	8.43774	28.1544	2.71811	0.85201	47.012	7.70512	4.5475	14.942	4.76689	11.3512	2.38125	0.65712	AIF1
49	BCAT1	6.96497	9.70958	16.6391	4.52299	5.18288	35.5351	7.00187	25.4846	4.49538	2.28899	50.9769	8.28483	4.73175	23.4525	5.25426	12.9374	2.46227	0.65226	BCAT1
50	COL8A2	8.87784	10.0663	20.907	10.5912	12.9531	59.9494	15.7418	49.9037	3.44522	4.21553	68.1372	10.1887	6.39401	20.4912	8.91397	18.9933	2.13074	0.64544	COL8A2
51	ANTXR1	8.57465	14.127	20.4434	6.9347	5.5856	60.6061	6.05036	21.6904	6.57746	1.02108	73.9245	9.75032	5.22111	23.418	6.07392	17.1671	2.82636	0.63986	ANTXR1
52	C10orf10	20.6264	37.3489	25.3657	16.4642	8.83772	43.116	21.0519	39.9026	3.951	5.20722	69.1082	19.0768	18.5762	23.7101	7.52883	20.2879	2.6947	0.6398	C10orf10
53	RECK	2036.57	1854.95	5268.47	1349.36	1165.08	9906.86	1536.32	6209.68	685.762	17.4083	17666.5	1913.34	2278.6	6209.28	1057.23	3610.41	3.41497	0.62724	RECK
54	TSPAN4	9.04622	12.603	41.8563	6.63457	10.2027	40.7793	13.6262	36.7008	3.69225	2.13777	74.8586	14.0578	11.6623	16.5698	8.10845	18.748	2.31216	0.61807	TSPAN4
55	SDC2	7.24969	13.7445	16.9232	4.55934	5.22101	34.2683	6.03379	24.5262	2.96953	5.38172	47.4227	10.7432	5.24909	18.1282	5.86201	12.7854	2.18106	0.61775	SDC2
56	EFEMP1	12.0177	8.28513	33.243	8.16078	7.27178	33.3434	13.9763	37.623	4.20556	5.4905	186.924	14.7767	48.4433	15.7185	19.499	31.0817	1.59401	0.61635	EFEMP1
57	SLC24A3	9.40948	7.49347	17.386	7.39804	34.0709	33.693	9.22309	48.1027	3.53807	2.41622	52.8493	10.8075	4.99324	19.5403	20.0406	17.5591	0.87618	0.61532	SLC24A3
58	FRZB	6.7647	7.08296	32.8557	6.74502	5.7934	32.5326	13.3595	22.736	2.61809	0.6838	51.3441	8.49595	4.55348	15.0909	5.5174	13.1534	2.38398	0.60505	FRZB

Table S1. CAFG (R≥0.6) expression, S/E ratio, and correlation coefficient in 28 BC cases of GSE10797
